# Acute Heart Failure in a Young Patient Treated in ICU—Diagnostic Pitfalls

**DOI:** 10.3390/clinpract14050155

**Published:** 2024-09-24

**Authors:** Łukasz Surówka, Paweł Andruszkiewicz, Monika Budnik, Robert Kowalik, Agnieszka Milner, Mateusz Zawadka

**Affiliations:** 1 2nd Department of Anaesthesiology and Intensive Care, Medical University of Warsaw, 02-097 Warsaw, Poland; 2 Department of Cardiology, Medical University of Warsaw, 02-097 Warsaw, Poland; 3 Department of Medical Microbiology, Medical University of Warsaw, 02-097 Warsaw, Poland

**Keywords:** Takotsubo syndrome, *Chlamydia pneumoniae*, cardiomyopathy, myocarditis

## Abstract

**Background:** *Chlamydia pneumoniae* and human herpesvirus 6 (HHV-6) are uncommon aetiological agents in respiratory tract infections and are rarely associated with cardiogenic shock. This case report presents a rare instance of severe cardiomyopathy linked to these infections in a 19-year-old Asian female. The case highlights the importance of considering a broad differential diagnosis in acute heart failure, especially in young adults. **Case report:** The patient was admitted with chest pain and diagnosed with ST-elevation myocardial infarction (STEMI) based on electrocardiography. She subsequently developed heart failure, with a marked reduction in myocardial contractility and a left ventricular ejection fraction (LVEF) of 20%. Treatment included broad-spectrum antibiotics and inotropic support guided by hemodynamic monitoring, leading to clinical improvement. The patient was discharged in a significantly improved condition following a stay in the intensive care unit (ICU). **Conclusions:** This case emphasizes the importance of considering Takotsubo syndrome in differential diagnoses, especially in ICU patients presenting with cardiogenic shock, to improve outcomes and reduce mortality through timely and appropriate management. Inotropic support, often used in the ICU to treat hypoperfusion, may worsen outcomes in patients with Takotsubo syndrome by exacerbating basal hypercontractility and prolonging the acute phase through catecholamine receptor activation.

## 1. Introduction

Many infectious agents can trigger inflammatory responses (e.g., myocarditis) and severely impair the heart (e.g., by reducing myocardial contractility, leading to heart failure). Acute heart failure in young adults requires a broad differential diagnosis, including bacterial infections, such as *Chlamydia pneumoniae*, or viral infections, such as HHV-6. A rare cause of heart failure is a *Chlamydia pneumoniae* infection, which typically spreads through respiratory droplets. In 90% of cases, a *Chlamydia pneumoniae* infection is asymptomatic or presents mild symptoms such as pharyngitis and laryngitis [1]. Severe complications like sepsis or cardiac shock due to *C. pneumoniae* are uncommon in clinical practice.

Additionally, the HHV-6 virus is a common cause of myocarditis, which can severely impact heart function. The clinical course of myocardial infection related to HHV-6 may vary depending on factors such as age, comorbidities, and immune response. In the initial phase, symptoms include flu-like signs (e.g., weakness, bone and joint pain, fever) often accompanied by respiratory tract inflammation. Most cases resolve spontaneously, but in some individuals, especially young patients, acute heart failure can develop. In addition to de novo infection, the reactivation of a latent infection is possible. HHV-6 can enter a latent state after a childhood infection, with heart inflammation occurring later due to viral reactivation [2,3].

## 2. Case Report

A 19-year-old Asian female with no recent travel history was admitted to the emergency department with severe chest pain and shortness of breath.

She reported a two-day history of symptoms, including a dry cough, nocturnal fever peaking at 39 °C, vomiting, dizziness, and near-syncope episodes. She denied any recent infections or chronic diseases and had no significant family history of cardiac conditions. Her medications included Escitalopram, which she had been taking for a year, and an emergency contraceptive pill taken eight days prior to admission.

Upon admission, the patient was alert and oriented, with no signs of lymphadenopathy or peripheral edema. On examination, she was tachycardic (130 beats per minute), with normal respiratory sounds and no pathological findings on abdominal examination. Initial vital signs showed hypotension (blood pressure 79/59 mmHg), normothermia (36.6 °C), and an oxygen saturation of 100% on room air. Electrocardiography indicated inferior wall ST-elevation myocardial infarction (STEMI), but subsequent coronary angiography revealed no abnormalities in the coronary arteries. Bedside echocardiography showed no significant contractility or valvular disease, and no pericardial fluid was noted. However, elevated D-dimer levels led to a CT scan, which ruled out pulmonary embolism, right ventricular strain, and aortic dissection.

**Figure 1:**
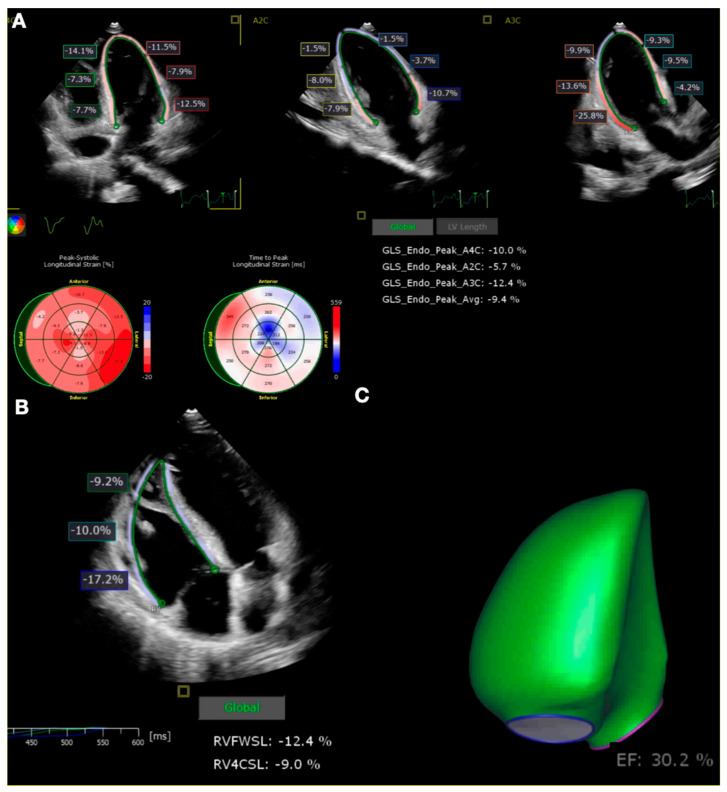
Global longitudinal strain assessment on transthoracic echocardiography. Global longitudinal strain of left ventricle on apical four-chamber, apical two-chamber, and three-chamber views (**A**), global longitudinal strain of right ventricle (**B**), and 3D reconstruction of right ventricle ejection fraction (**C**).

Abnormalities in the laboratory tests included the following: Troponin-I: 19,437 ng/L [N < 60 ng/L], NT-proBNP: 15,352 pg/mL [N < 125 pg/L], CRP: 17.8 mg/L [N < 10 mg/L], Lactates: 11.7 mmol/L [N < 1.6 mmol/L]. The next day, the patient’s condition deteriorated. Another echocardiography revealed a hypokinesis of the left ventricle, with an ejection fraction (LVEF) of 20%. Global longitudinal strain (GLS) of the left ventricle was −9.4%. Right ventricular dysfunction was observed, and the right ventricle ejection fraction (RVEF) was 30.2%. GLS of the free wall of the right ventricle was −9.0% (Figure 1). Subsequently, the patient developed respiratory failure, hemodynamic instability, liver failure and acute kidney injury, necessitating a transfer to the intensive care unit.

## 3. Intensive Care Unit Admission and Management

**Figure 2:**
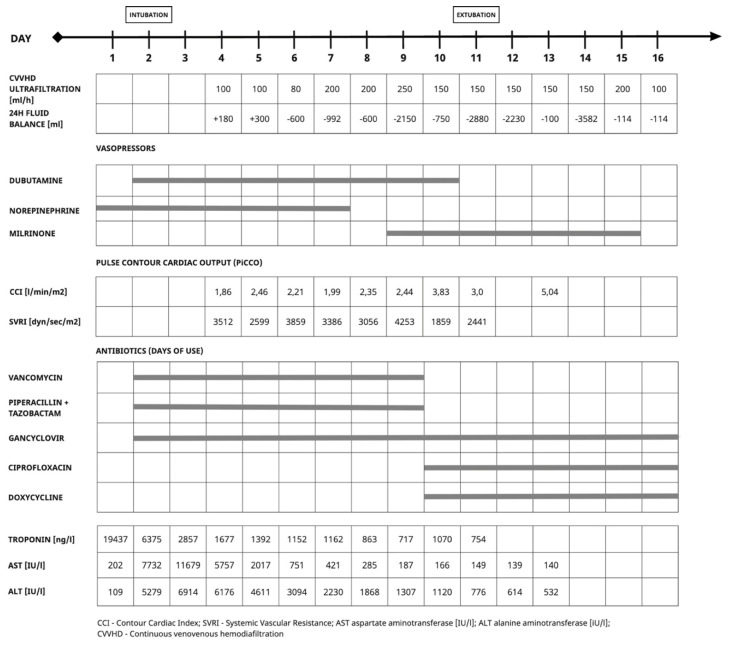
Caption. Management during ICU stay.

Upon admission to the intensive care unit (ICU), the patient was alert but exhibited signs of somnolence. She was breathing spontaneously and required passive oxygen therapy via a non-rebreather mask. Significant circulatory failure soon developed, as evidenced by hypotension (blood pressure 75/40 mmHg), tachycardia (heart rate 109/min), and anuria (Figure 2).

The following day, intubation and mechanical ventilation became necessary due to the development of respiratory failure. Hemodynamic support was initially provided with a dobutamine infusion, titrated to the desired effect. However, persistent circulatory instability, characterised by low cardiac output and high systemic vascular resistance, led to the initiation of milrinone, with the discontinuation of dobutamine. Continuous veno-venous hemodiafiltration (CVVHD) was initiated, with a high ultrafiltration rate aiming for a negative fluid balance to offload the right ventricle. Over the subsequent days, the patient’s condition gradually stabilised.

Elevated inflammatory markers (CRP and procalcitonin) prompted empirical antimicrobial therapy with vancomycin and piperacillin with tazobactam. A positive polymerase chain reaction (PCR) result for human herpesvirus 6 (HHV-6) necessitated the addition of ganciclovir. Coagulopathy due to liver failure precluded the performance of myocardial biopsy at that time.

Microbiological assays showed positive IgM serologies for *Chlamydia pneumoniae* and a positive HHV-6 test. Consequently, the antimicrobial regimen was modified to include doxycycline and ciprofloxacin. Broad laboratory and microbiological diagnostics were conducted during hospitalisation, revealing positive results for *Chlamydia pneumoniae* IgM (+) and IgG (+) and HHV (+). Tests for vascular and rheumatological diseases were negative, and cerebrospinal fluid examination was unremarkable. Over the following days, the patient’s hemodynamic and respiratory function gradually improved, enabling weaning from mechanical ventilation and the discontinuation of sedation and hemodynamic support. A follow-up transthoracic echocardiogram showed improved left and right ventricular contractility, with an LVEF of 53%, GLS LV of −18.6%, RVEF of 48.4%, and GLS RV of −30%. Follow-up testing for *Chlamydia pneumoniae* indicated seroconversion. The patient was subsequently discharged from the ICU in stable condition.

After being transferred from the ICU, the patient was admitted to the cardiology department, where renal replacement therapy was continued, and her treatment regimen was gradually reduced. She remained hospitalised for two months, with her primary challenges being significant muscle weakness and other symptoms associated with post-ICU syndrome, such as cognitive impairment, difficulty concentrating, and anxiety. Upon discharge, she was taken by her parents to India.

## 4. Discussion

New onset heart failure in a young patient requires a broad differential diagnosis. The above case describes a patient with a fulminant course of cardiogenic shock of unknown aetiology with an acute impairment of systolic function of both.

In this case, the differential diagnosis included a range of bacterial infections, with a focus on atypical pathogens such as *Chlamydia pneumoniae*, *Mycoplasma pneumoniae*, and *Chlamydia trachomatis*. Concurrently, assessments for viral infections were conducted. Additionally, diagnostic evaluations for vascular and rheumatological diseases were performed. To determine the underlying cause of the severe cardiomyopathy, septic, autoimmune, viral, and toxic aetiologies were all considered. Takotsubo syndrome was suspected due to the rapid and complete recovery of heart contractility in the absence of any coronary vessel occlusion. Takotsubo syndrome affects approximately 2% of patients who present with chest pain and elevated troponin levels. Over 90% of these patients are women, predominantly postmenopausal. The majority report classic symptoms, including chest pain, shortness of breath or dyspnoea, syncope, and palpitations. Older patients tend to have a poorer prognosis due to the presence of complicating factors such as stroke, severe arrhythmias, acute heart failure, and cardiogenic shock [4]. Significant changes in the ECG and arrhythmias are common, yet coronary angiography often reveals normal coronary arteries [5,6].

The pathophysiology is not fully understood. The main cause is the occurrence of significantly elevated levels of catecholamines and stress-related neuropeptides in plasma. Takotsubo syndrome is often induced by mental or physical stress. This theory is supported by clinical observations indicating that this condition is most frequently diagnosed in people who report the action of a stressor in their history.

Takotsubo syndrome is an acute, severe, usually transient left ventricular dysfunction. The latest reports indicate an increased mortality rate comparable to the incidence of patients with acute coronary syndrome [5,6]. A large proportion of patients develop cardiogenic shock [7]. Early identification by echocardiography is crucial and has important implications in selecting appropriate treatment. Up to 20% of patients admitted to the ICU may suffer from secondary Takotsubo syndrome, which may result from trauma, infection, post-cardiac arrest, respiratory failure, and surgery [5].

However, it seems that the above case does not meet all the criteria for Takotsubo syndrome. A myocardial biopsy was not performed to exclude myocarditis due to severe coagulation disorders. Additionally, a positive HHV-6 result was identified. HHV-6 is a significant and common cause of myocarditis, which can severely impact cardiac function. Before hospital admission, the patient exhibited inflammatory symptoms, including cough, fever, vomiting, and chills. These symptoms support an inflammatory aetiology for the severe cardiomyopathy observed, with subsequent resolution and improvement in symptoms, including the impaired systolic function of the left ventricle. The fulminant course of cardiogenic shock, followed by relatively rapid clinical improvement after broad-spectrum treatment encompassing both antiviral and antibacterial therapies, suggests that the patient’s condition may not be attributed to a single cause. It is more likely that multiple factors and their interaction contributed to the patient’s severe state.

It is important to recognise that Takotsubo syndrome is frequently suspected in patients admitted to the ICU. Many ICU patients are treated with catecholamines to manage systemic hypoperfusion, but this can be harmful in cases of Takotsubo syndrome. Positive inotropic agents may exacerbate basal hypercontractility, potentially prolonging or worsening the acute phase of the syndrome by activating catecholamine receptors or their downstream molecular pathways. Furthermore, catecholamine infusion has been associated with poorer in-hospital outcomes and higher long-term mortality rates compared to patients who do not receive catecholamine support. Therefore, it might be prudent to consider using catecholamine-sparing agents, such as vasopressin and milrinone. Levosimendan can also be an option and, in the acute phase, beta-blockers may be considered to block the effects of adrenergic surges. These alternatives can help mitigate the adverse effects associated with catecholamine use in patients with Takotsubo syndrome [6,7].

The patient’s use of escitalopram and contraception, as well as an in-depth interview about her episodes of depression, may constitute a stress factor for the patient and thus contribute to the occurrence of Takotsubo syndrome. Research has shown that venlafaxine and fluoxetine are significant risk factors; however, this finding needs to be confirmed via large epidemiological studies [8,9].

The literature and scientific research have highlighted individual cases of severe and fulminant cardiogenic shock, though these remain rare [10,11,12]. Most patients die due to the severity of their complications. Long-term rates of overall mortality and recurrence are not trivial, and some presenting features (e.g., older age, physical stressor) were significantly associated with an unfavourable long-term prognosis [13,14].

## 5. Conclusions

This case highlights the necessity of individualising the treatment and diagnostics for each patient. Frequently, obtaining a clear diagnosis is challenging. ICU patients often present with a convergence of risk factors and complications, exhibiting characteristics that align with multiple potential diagnoses. Therefore, a tailored approach is essential to effectively address the complex and multifaceted nature of their conditions.

## Data Availability

The original contributions presented in the study are included in the article.
